# Strength of Composite Pressure Insulators for High Voltage Circuit Breakers: An Experimental and Numerical Investigation

**DOI:** 10.3390/ma17112741

**Published:** 2024-06-04

**Authors:** Jan Ferino, Gabriela Loi, Andrea Meleddu, Francesco Aymerich, Iuri Mazzarelli, Elisa Pichini

**Affiliations:** 1 Astarte Strategies s.r.l, Via San Saturnino 7, 09124 Cagliari, Italy; a.meleddu@astarte-strategies.com; 2 Department of Mechanical, Chemical, and Materials Engineering, University of Cagliari, Via Marengo 2, 09123 Cagliari, Italy; gabriela.loi@unica.it; 3 INAIL—Istituto Nazionale per l’Assicurazione contro gli Infortuni sul Lavoro, ICVR UOT, 20123 Milano, Italy; i.mazzarelli@inail.it; 4 INAIL—Istituto Nazionale per l’Assicurazione contro gli Infortuni sul Lavoro, DIT, 00143 Roma, Italy; e.pichini@inail.it

**Keywords:** composite pressure insulators, split-disk testing method, hoop strength, FE modeling

## Abstract

Glass fiber-reinforced composite cylinders, capable of withstanding internal pressure generated during service, are increasingly utilized as insulators in high voltage circuit breakers. Different testing procedures have been suggested by various standards to assess the pressure resistance of these components. Due to its simplicity and cost-effectiveness, the split-disk testing method is the most widely used for evaluating the hoop strength of pressure cylinders during the development and verification phases. However, the method presents several aspects, such as those related to the influence of specimen geometry and friction, which require further examination since they may impact the outcome of the experimental tests. The investigation, carried out by a combination of experimental testing and finite element analyses, shows that the friction between the specimen and the semi-disks has a noteworthy effect on the hoop load applied to the specimen. Almost constant load distributions along the hoop direction, representative of the real operating conditions in a pressurized cylinder, can be achieved via proper lubrication of the contact surfaces. Furthermore, FE analyses demonstrate that the notch geometry suggested by specific standards (short notch) is not capable of inducing a uniform strain distribution in the notched region. A different notch geometry (long notch) is proposed in the study to attain a more uniform strain field over the reduced area region. The experimental results indicate that the strength measured on the short notch specimens is higher than that determined on the long notch specimens, thus confirming the significant influence of strain distribution on the strength properties measured with the split-disk method.

## 1. Introduction

High voltage circuit breakers, designed to interrupt the flow of electricity in case of overloads or faults, are frequently installed in strategically important power distribution systems and networks to protect equipment and infrastructure from damage. Hollow insulators that contain a special dielectric gas capable of extinguishing the electrical arc are typically integrated into high voltage circuit breakers [1]. The insulators are designed to withstand the pressure buildup generated during arc interruption and to prevent leakage of the gas into the environment.

**Figure 1:**
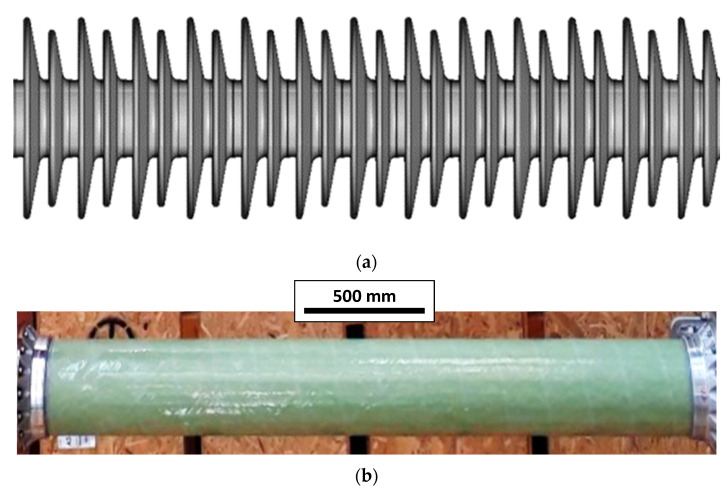
(**a**) High voltage circuit breaker and (**b**) glass fiber-reinforced composite insulator.

Glass fiber-reinforced composite insulators (Figure 1) are increasingly replacing traditional ceramic counterparts in high voltage circuit breakers due to their higher specific strength, damage tolerance, design flexibility, and lower susceptibility to pollution effects. Composite insulators are typically manufactured by filament winding as cylindrical elements, which are subsequently cut to size according to the desired length [2]. Filament winding offers several advantages over alternative technologies, including high fiber volume fractions, reduced void content, and the ability to enable cost-effective process automation while ensuring high accuracy in fiber alignment [3].

Specific test methods and acceptance criteria for pressurized insulators for use in high voltage electrical equipment, as well as general recommendations for their design and construction, are provided by the international IEC 61462:2007 standard [4]. Additional rules or guidelines regarding the manufacturing and testing of gas pressure vessels containing active parts of electrical equipment also exist at a national level. As an example, the Italian VSR.8.B.2 rule [5] provides the technical specifications for the construction and use of vessels made of elements, including both insulating and metal materials. With reference to the required pressure burst test, the minimum testing pressure ($p_{r}$) that should be sustained by the component is given by Equation (1):

(1) $$p_{r}=4.25\cdot \frac{R}{R_{m}}\cdot p$$

where R and $R_{m}$ represent, respectively, the average and minimum hoop strengths of the material, which must be certified by the component manufacturer via a series of mechanical tests. Therefore, although a full-scale burst test on the entire component is mandatory for the qualification process, various alternative testing approaches have been proposed for evaluating the hoop strength of wound composite insulators without performing costly and complex tests during the development phase.

While flat rectangular or dog-bone samples are suggested in the most widely adopted recommendations for composite materials, the properties obtained from composite plates manufactured by winding reinforcing fibers over a flat mandrel cannot be considered truly representative of those that characterize the performance of filament-wound cylindrical components [6]. Due to the strong dependence of composite strength on manufacturing technology, specimens directly extracted from the insulators should therefore be used to determine properties that describe the actual behavior of the component. Ring specimens obtained by cutting the cylindrical pressure component perpendicular to its axis are often used to characterize the hoop (i.e., circumferential) strength of the material, and several testing approaches have been proposed to introduce in the specimen a reasonably uniform tensile stress state.

A very simple testing setup, where the specimens are loaded using a three-point bending scheme, was used in [7] to study the fracture resistance of ring specimens extracted from plastic pipes. However, the chosen three-point loading configuration leads to a bending stress state very different from the hoop tensile stress experienced by the pipes in service.

In burst tests, the hoop stress in the ring specimens is generated either by internal pressurized fluid [8,9] or by inserting a tapered rod into a multi-piece or monolithic system that expands radially on the tested ring [10,11,12]. However, these test methods require dedicated equipment (pressurizing pump, anti-leakage seals, or rubber bladders) and special preparation of the specimen or the use of complex loading fixtures, and are therefore rather expensive, time-consuming, and difficult to implement.

The standardized split-disk method [13,14,15] is probably the testing approach most frequently adopted to evaluate the hoop tensile strength of ring specimens cut from cylindrical pressure systems. In these tests, the specimen is loaded to failure by the surfaces of two semi-disks, which are pulled apart by a testing machine, thereby inducing hoop stress in the wall of the specimen. This method has, however, the disadvantage of introducing undesired bending moments and resulting stress concentrations in the regions of the specimen between the two semi-disks [16]. These local stress perturbations increase with the application of the load and the separation of the disks, ultimately leading to premature failure in the sectors of the specimen unsupported by the semi-disks.

Even though complex testing devices consisting of multiple disk segments [16,17] have been proposed to mitigate this issue, a simpler and more frequently adopted strategy involves the use of notched specimens, as suggested by the ASTM D2290, EN 1394, and ISO 8521 standards [13,14,15]. The insertion of a notch in the specimen at a distance from the edges of the semi-disks reduces the cross-sectional area of the specimen, thereby ensuring that failure occurs away from the region with the bending-disturbed stress field.

As an example, the EN 1394 standard specifies that the cross-sectional area of the ring specimens should be reduced by machining two circular notches, each 10 mm in radius, which decreases the width of the ring from 25 mm to 15 mm. Similar notches and ring widths are suggested by the ASTM D2290 standard, which indicates notches with a minimum radius of 0.35″ (∼8.9 mm) for a ring with a minimum width of 0.9″ (~22.9 mm). When testing reinforced thermosetting materials, the standards specify that the ring specimen should be mounted on the test fixture so that the notched area is away from the split in the fixture and fully supported by the surfaces of the disk. In particular, an angle of 10° or a distance of two inches between the disk edge and the reduced area are, respectively, suggested by the EN 1394 and ASTM D2290 standards.

However, despite the guidelines provided by the standards, there is no general consensus on the use of notched ring specimens in split-disk tests to evaluate the hoop strength of composite pressure components, as evidenced by the different testing approaches proposed across various studies.

Unnotched specimens were chosen in [6,18,19,20,21,22] to characterize the effect of manufacturing parameters, material systems, and winding layups on the hoop tensile properties of filament-wound tubes. The studies indicate that the strength of the rings is affected by the local bending that occurs at the gap between the semi-disks, with the ultimate failure taking place in the region of the rings unsupported by the disks. It is worth remarking that the adoption of unnotched ring specimens could be strictly required when the components are manufactured with low winding angles (i.e., with all fibers aligned at angles close to 0° with respect to the circumferential direction) to avoid early matrix splitting at the notch parallel to the fiber direction [23].

Ring specimens notched according to the recommendations provided in [13,14,15] were adopted in [24,25,26,27,28,29,30] to evaluate the hoop properties of tubular composites using the split-disk testing method. However, in many of the studies [24,25,26,28,29], the ring specimens were mounted on the test device with the notched sections positioned across the split of the fixture. The failure thus occurred in the unsupported region of the ring, which is characterized by a stress field greatly affected by spurious local bending. Conversely, the tests reported in [27,30] followed the specific guidelines of the ASTM D2290 standard, with the reduced areas centered away from the split of the fixture.

Another issue with the split-disk test concerns the friction that develops between the specimen and the surface of the disks during the application of the load [18,20,22]. As a result of this friction, the hoop load sustained by the supported portions of the specimen is lower than that applied by the testing machine to the loading fixture. This difference increases with the distance from the split of the fixture due to the cumulative effect of friction forces over an increasingly wider arc. Liquid lubricants [6], grease [18], or graphite powder [22] applied to the disk surfaces, or even needle rollers inserted between the specimen and the disks [20], have been used to reduce the influence of friction on the outcomes of the split-disk tests.

A further issue of the split-disk method, which, according to the authors, has not been adequately examined in the existing literature, concerns the reduction in the cross-sectional area of the specimen, which is commonly achieved via two circular notches machined at the sides of the specimens [13,14,15]. However, the recommended notched geometry does not seem suitable for generating a region with a stress distribution uniform enough to represent the service response of the real component.

The aim of the study is therefore to assess, via experimental and numerical investigation, the impact of key aspects related to the practical implementation of the testing approach, such as notch geometry and friction, on the reliability and accuracy of split-disk test results. In particular, a novel geometry of notched specimens, capable of ensuring a more uniform stress distribution than that of standardized specimens, is proposed and examined in this paper.

## 2. Materials and Experimental Methods

The ring specimens used for the tests were cut with a diamond blade from insulators provided by *Hitachi Energy*, which are utilized in 145/170 kV high voltage circuit breakers. The insulators were manufactured using a filament winding process with E-glass fibers and epoxy resin and featured a $\left[\pm 30_{2}/\pm 68_{4}/\pm 30\right]$ layup (the 0° angle corresponds to the hoop direction), with an internal diameter of 154 mm and a wall thickness of 6 mm.

**Figure 2:**
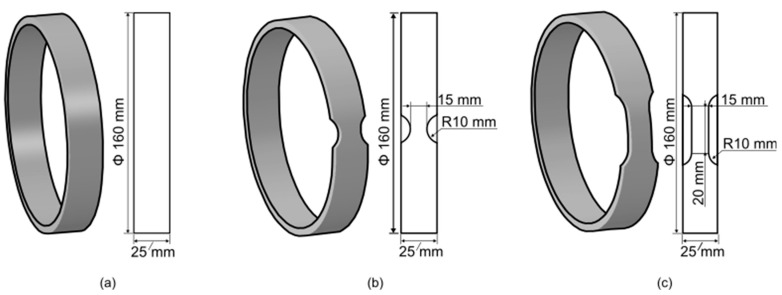
Geometry of the tested specimens: (**a**) unnotched specimen, (**b**) short notch specimen, and (**c**) long notch specimen.

Both unnotched and notched specimens, with two types of notch geometries to create regions with reduced areas (Figure 2), were tested. In the first notch geometry (namely, “short notch”), the cross-sectional area was reduced via two circular notches with a radius of 10 mm, following the recommendations of the EN-1394 standard. In the second notch geometry (namely, “long notch”), the transition between the full area and the reduced area is still achieved via a radius of 10 mm, but the region with the constant reduced area has a gauge length of 20 mm. The width of all specimens in the full area was 25 mm, while the reduced width of both types of notched specimens was 15 mm. Geometries and dimensions of the examined ring specimens are shown in Figure 2.

**Figure 3:**
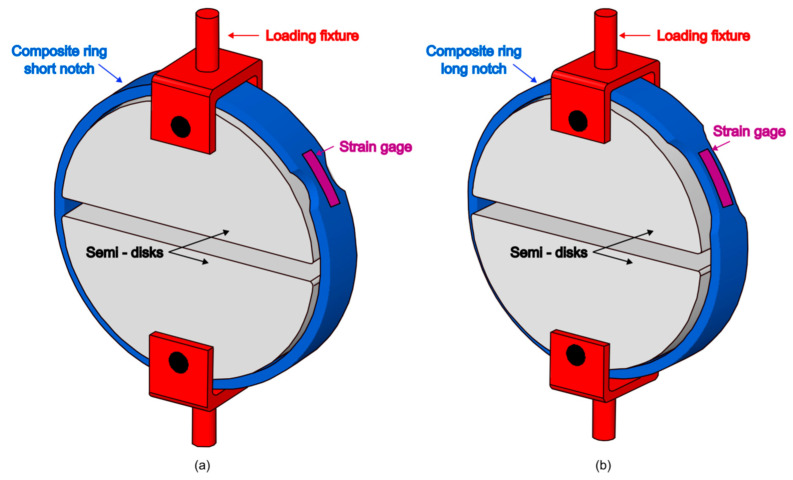
Testing fixture and notched geometries: (**a**) short notch specimen and (**b**) long notch specimen.

The tests were conducted on a servo-electric *INSTRON 5585H* testing machine, using a split-disk loading fixture as described in the EN 1394 standard (Figure 3) and equipped with 35 mm wide steel semi-disks having a diameter of 153 mm (as compared to an internal diameter of 154 mm for the specimens). The external surface of the semi-disk was lubricated with industrial grease. Tests were performed under displacement control at a rate of 4 mm/min, with an initial gap between the two semi-disks of 6 mm. All the specimens were centered across the width of the semi-disks to prevent any potential out-of-plane bending on the fixture.

To avoid the influence of local bending resulting from the straightening of the unsupported region between the semi-disks, the notched specimens were positioned so that the entire region with the reduced cross-sectional area was supported by the surface of the semi-disks, as illustrated in Figure 3. Strain gauges with a grid length of 20 mm were bonded to notched specimens to monitor the strain in the reduced area region.

## 3. Experimental Results

**Figure 4:**
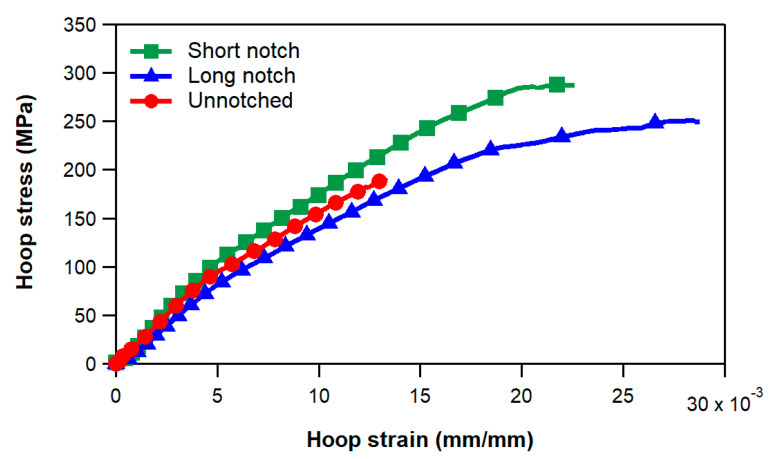
Experimental hoop stress–strain curves.

**Figure 5:**
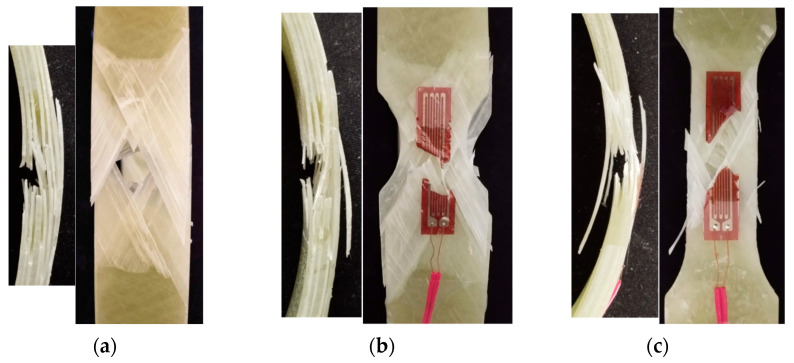
Failure appearance of the specimens: (**a**) unnotched specimen, (**b**) short notch specimen, and (**c**) long notch specimen.

Figure 4 presents typical experimental stress–strain curves of unnotched and notched specimens. As suggested by the relevant standards [13,14], the hoop stress was calculated using the following equation:

(2) $$\sigma =\frac{P}{2A}$$

where P is the load applied to the testing fixture, and A is the minimum cross-sectional area of the ring specimen. As specified in the previous section, the strain values were measured by 20 mm strain gauges bonded to the specimens at the locations indicated in Figure 3. The average strengths and the failure appearances of the three types of specimens are, respectively, reported in Table 1 and shown in Figure 5.

We can readily notice that the strength of the unnotched rings is notably lower than that of the notched specimens. This outcome was expected, as the failure of unnotched rings is primarily controlled by bending and strain concentration effects occurring at the unsupported length of the specimens. This is clearly evidenced by the location of the failure, shown in Figure 5a, which always occurred at the gap between the semi-disks of the fixture. These results indicate that the introduction of the notch at a position away from the split of the fixture is strictly necessary for a reliable evaluation of the hoop strength of the composite rings.

With regard to the notched specimens, the experimental tests show that the strength of the short notch specimens (i.e., with the notch geometry suggested by the ASTM and EN standards) is higher than that of the long notch specimens. Both types of notched specimens, after a short initial nonlinearity stage due to the gradual settling of the specimen on the disks, exhibit a linear hoop stress vs. strain trend up to a stress of about 100 MPa (Figure 4). Above this level, the stress–strain curves present a progressive slope decrease until final failure. The knee of the stress–strain curves was visually observed to correspond to the onset of matrix cracks, which develop at the termination of the circular transition regions, first in the ±30° layers and then in the ±68° layers. With increasing loads, the matrix cracking propagates across the reduced area region, eventually leading to the final failure of the rings. No delaminations were observed to develop between the layers before the failure of the specimens. Post-mortem inspections of the fracture surface show that the ultimate failure occurs by separation of adjacent layers and without extensive fiber fracture. The debonding between layers with different orientations is promoted and triggered by the dense and diffuse matrix cracking that spreads across the notched region (Figure 5a,b) before specimen failure.

To investigate the stress distribution and associated damage progression within the composite rings and explore possible reasons for the difference in strength between the short notch and long notch specimens, detailed finite element analyses have been performed on the behavior of the composite rings when loaded through the split-disk setup.

## 4. FE Model

A 3D finite element model of the composite ring was developed using the commercial finite element (FE) method package ABAQUS (Dassault Systemes, Vélizy-Villacoublay, France) to investigate the stress distribution within the notch region and the occurring damage mechanisms. For this purpose, three finite element models, individually corresponding to the three considered specimen geometries, were built to reproduce the adopted experimental set-up, consisting of two cylindrical semi-disks in contact with the internal surface of the specimen (Figure 3).

The semi-disks were modeled as discrete analytical rigid surfaces. In contrast to rigid surfaces formed by element faces, analytical rigid surfaces provide a more accurate representation of the physical contact and a smoother surface description, reducing the number of contact tracking operations and, thus, improving the contact algorithm performance [31]. Each semi-disk was associated with a reference point representing the location at which the boundary conditions were applied, and the resulting reaction force was acquired.

The composite ring was modeled as a 3D deformable continuum solid body and discretized using 8-node hexahedral continuum shell elements (SC8R) with reduced integration and enhanced hourglass control. The stacking sequence of the composite ring was discretized using one element per layer. A sensitivity analysis was preliminary performed to determine the appropriate element dimensions for results to be independent of the mesh. The ring was finally discretized employing 0.5 mm × 0.5 mm elements with a mesh refinement of 0.3 mm × 0.3 mm within the notched areas.

In order to accurately simulate the mechanical response of the composite ring, an orthotropic linear-elastic model was combined with the Hashin criterion for predicting the initiation, propagation, and interaction of the occurring damage modes. Based on Hashin’s theory [32,33], the selected damage model considers four damage initiation mechanisms: matrix tension, matrix compression, fiber tension, and fiber compression. The associated initiation criteria can be expressed as

(3) $$F_{fiber\;tension}={\left(\frac{\sigma_{11}}{X_{t}}\right)}^{2}+\alpha {\left(\frac{\sigma_{12}}{S_{l}}\right)}^{2}$$

(4) $$F_{fiber\;compression}={\left(\frac{\sigma_{11}}{X_{c}}\right)}^{2}$$

(5) $$F_{matrix\;tension}={\left(\frac{\sigma_{12}}{Y_{t}}\right)}^{2}+{\left(\frac{\sigma_{12}}{S_{l}}\right)}^{2}$$

(6) $$F_{matrix\;compression}={\left(\frac{\sigma_{22}}{{2S}_{t}}\right)}^{2}+\left[{\left(\frac{Y_{c}}{2S_{t}}\right)}^{2}-1\right]\frac{\sigma_{22}}{Y_{c}}+{\left(\frac{\sigma_{12}}{S_{l}}\right)}^{2}$$

where $\sigma_{ij}$ indicate the effective stress components, $X_{t}$ and $X_{c}$ are the tensile and compressive strength along the fiber direction, $Y_{t}$ and $Y_{c}$ represent the tensile and compressive strength in the transverse direction, $S_{l}$ and $S_{t}$ denote the longitudinal and transverse shear strengths. The coefficient α, which quantifies the contribution of the longitudinal shear stress to the fiber tensile failure, was set to 0, as specified in the model proposed in Hashin and Rotem [33].

As soon as an initiation condition is satisfied, damage occurs, and any increase in the applied load causes the degradation of the material stiffness. The reduction in the stiffness coefficient (i.e., material softening) relies upon the model of Matzenmiller et al. [34], in which a set of damage variables was defined to correlate the damaged stress tensor with the stress components acting in undamaged conditions. Thus, the stiffness matrix of the failed material can be written as follows [35]:

(7) $$C=\frac{1}{D}\;\left[\begin{array}{ccc} (1-d_{f})E_{1} & (1-d_{f})(1-d_{m})\nu_{12}E_{1} & 0\\ (1-d_{f})(1-d_{m})\nu_{12}E_{1} & (1-d_{m})E_{2} & 0\\ 0 & 0 & D\;\left(1-d_{s}\right)G_{12} \end{array}\right]$$

(8) $$D=1-\left(1-d_{f}\right)\;\left(1-d_{mt}\right)\;\left(1-d_{mc}\right)$$

where $E_{1}$ and $E_{2}$ are the elastic moduli in the fiber and transverse direction of the undamaged material, $\nu_{12}$ and $\nu_{21}$ the Poisson ratios, and $G_{12}$ the shear modulus. Similarly, the term $d_{s}$ represents the damage variable associated with shear, whose expression is a function of the other damage variables:

(9) $$d_{s}=1-\left(1-c\right)\left(1-d_{fc}\right)\nu_{12}\nu_{21}$$

Values of the damage variables range between zero (undamaged condition) and 1 (fully damaged conditions).

After the damage onset, the damage evolution law relies upon the definition of equivalent displacement to reduce the dependence of damage evolution on mesh density ([31,36]). The law governing the growth of each damage variable can thus be expressed as follows [35]:

(10) $$d_{I}=\frac{\delta_{I,eq}^{f}\;(\delta_{I,eq}-\delta_{I,eq}^{0})}{\delta_{I,eq}\;(\delta_{I,eq}^{f}-\delta_{I,eq}^{0})}\;\;\;\;\;\;\;I\;\in \;\left\{ft,\;fc,\;mt,\;mc\right\}$$

with $\delta_{I,eq}^{0}$ representing the equivalent displacement corresponding to the damage onset and $\delta_{I,eq}^{f}$ indicating the equivalent displacement at which the material completely fails. The latter quantity can be obtained as the ratio between the critical energy release rate ($G_{IC}$) for the considered damaged mode and the equivalent stress at which the associated damage initiation criterion is met ($\sigma_{I,eq}^{0}$):

(11) $$\delta_{I,eq}^{f}=\frac{2G_{IC}}{\sigma_{I,eq}^{0}}\;$$

The main elastic and fracture properties of the material used in the analyses are listed in Table 2 ([37,38,39,40,41,42]).

The contact interaction between the composite ring and the two semi-disks was simulated by a surface-to-surface contact pair, with the outer surface of the rigid disks being the master surface and the inner surface of the ring acting as the slave surface. A hard pressure-overclosure response enforced by a penalty approach and a Coulomb friction model with a friction coefficient of 0.1 were applied to describe the normal and tangential behavior of the interacting surfaces, respectively. Several simulations were also run by varying the friction coefficient value between 0.05 and 0.2. Since a thin film of lubricating grease was applied on the semi-disks before the experimental tests, this range well represents the friction between the ring and the metallic surface [43]. In order to simulate the test conditions, the lower semi-disk was completely fixed, while the upper disk was constrained to only move upward to reproduce the crosshead displacement of the tensile machine.

## 5. Results and Discussion

**Figure 6:**
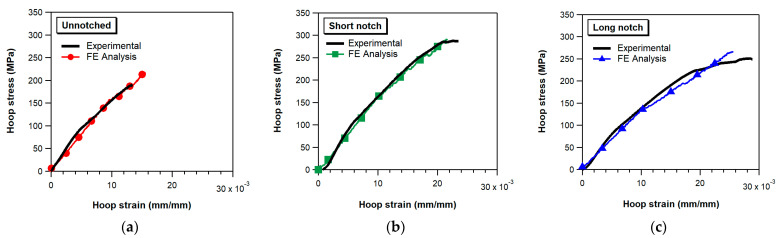
Comparison between experimental and numerical hoop stress–strain curves: (**a**) unnotched specimen, (**b**) short notch specimen, and (**c**) long notch specimen.

Figure 6 presents a comparison between experimental and numerical hoop stress–strain curves (calculated for a friction coefficient of 0.1) for both unnotched and notched specimens. The strain values displayed on the plots were determined as the average of strains within a region of the FE model corresponding to the grid area of the strain gauges bonded to the specimens. The hoop stress of the FE curves was derived by dividing the calculated constraint force applied to the semi-disks by the cross-sectional area (cross-sectional reduced area in the case of notched specimens).

We observe a reasonably good agreement between the numerical results and the experimental data for all three types of ring specimens analyzed. Particularly, we note that the FE model simulates the progressive decrease in slope observed for stress values larger than about 100 MPa in all ring specimen configurations. The FE analyses also capture the difference in hoop strength among the various specimen geometries, with unnotched samples exhibiting a significantly lower strength than notched specimens and long-notched specimens showing a lower strength compared to short notch ones.

**Figure 7:**
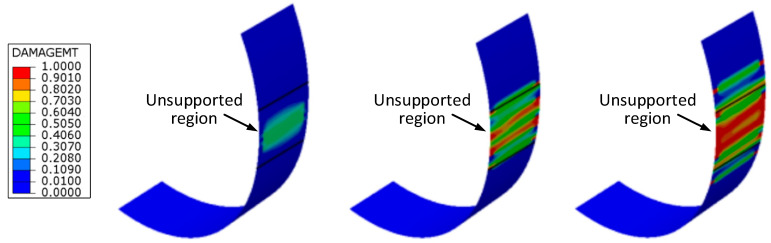
Matrix damage maps predicted by the FE analyses at increasing loads for the unnotched specimen. Colors represent the value of the damage parameter *d_m_* of the +30° layer closest to the internal diameter of the ring specimen.

The initiation and progression of the damage modes accumulating before ultimate failure are also properly simulated by the FE calculations. The damage maps predicted for the unnotched ring specimen at different steps of the loading history (Figure 7) show that the damage initiates in the form of matrix cracks at the unsupported length of the ring due to stress concentration and bending effects at the edges of the semi-disks. The final failure is predicted by the FE model to occur when the matrix cracks spread over the entire unsupported region of the ring specimen, as observed in the experiments (Figure 5).

**Figure 8:**
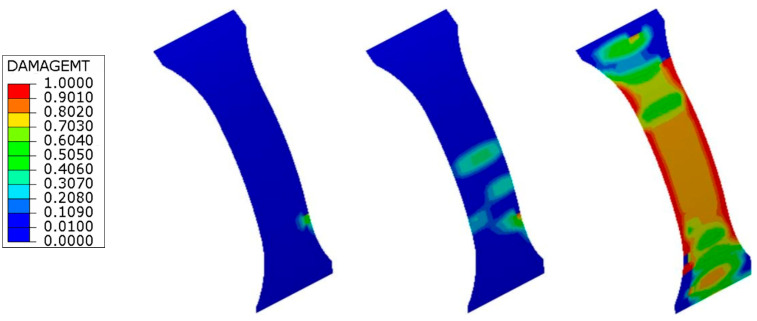
Matrix damage maps predicted by the FE analyses at increasing loads for the long notch specimen. Colors represent the value of the damage parameter *d_m_* of the +30° layer closest to the internal diameter of the ring specimen.

**Figure 9:**
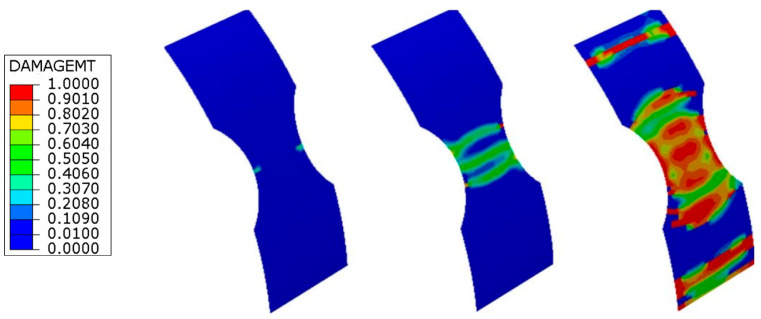
Matrix damage maps predicted by the FE analyses at increasing loads for the short notch specimen. Colors represent the value of the damage parameter *d_m_* of the +30° layer closest to the internal diameter of the ring specimen.

Figure 8 shows the evolution of damage developing in the long notch ring specimens. Consistent with experimental observations, the FE analyses predict that matrix cracking damage initiates in the ±30° layers, starting at the end of the curved transition region leading to the narrower part of the specimen. The onset of this matrix damage is also correctly simulated at a stress level that corresponds to the onset of stiffness decrease visible in the experimental stress–strain curve. Under increasing load, the matrix damage predicted by the FE model also develops in the ±30° layers close to the internal surface of the ring and in the central ±68° layers, finally spreading across the entire reduced area region and ultimately leading to separation of the specimen at the notch ligament. A similar sequence is provided by the FE simulations for the damage response of the short notch specimens (Figure 9).

The influence of friction between the semi-disks and the specimen on the hoop load sustained by the specimen wall was explored by examining the results of the FE analyses for different friction coefficient values.

**Figure 10:**
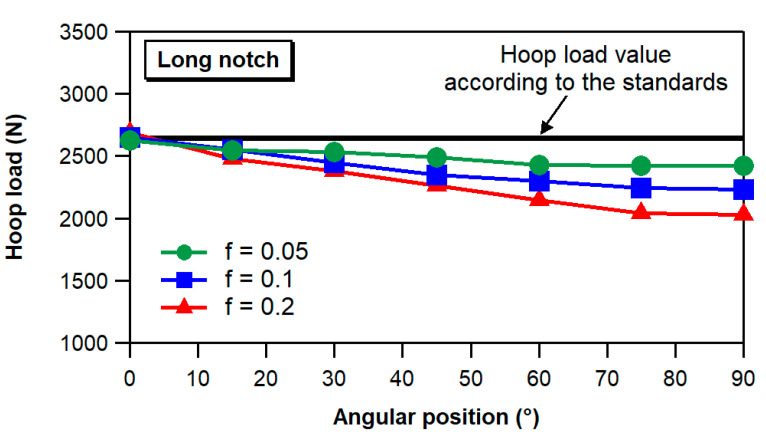
Hoop load as a function of the angular position for different friction coefficient values (load applied to the modeled testing fixture = 5300 N). The black line represents the hoop load calculated according to the indications of the ASTM and EN standards.

If we focus our attention on the value of the hoop load as a function of the angular position along the semi-disk, we may notice that, due to friction forces, the load actually carried by the ring wall generally decreases when moving away from the unsupported region at the split of the fixture. Therefore, the hoop load at the notch is lower than the hoop load calculated as suggested by the ASTM and EN standards (half of the load applied to the loading system). In particular, as shown in the graph of Figure 10, the hoop load exhibits a significant decrease with increasing angular position for friction coefficients higher than approximately 0.2. Conversely, low friction coefficients result in almost constant values of the hoop force across the full range of circumferential angular positions. However, it is worth noting that the hoop force remains significantly lower than the value suggested by the standards, even for very low friction values.

These findings indicate that uniform hoop load distributions, such as those occurring in pressurized insulators, can be achieved by ensuring low friction between the loading system and the composite ring under test. It is worth mentioning that friction coefficients lower than 0.2 are easily achieved via appropriate lubrication between steel- and glass-reinforced polymers [43], particularly when dealing with contacts between smooth surfaces. These results not only highlight the critical importance of lubrication but also suggest particular care when determining the hoop strength of composite rings based on the measurement of the load applied by the testing machine to the loading fixture. As an example, the graph of Figure 10 indicates that at an angular position of 45°, a difference of about 15% exists between the value of the hoop force recommended by the ASTM and EN standards and that calculated via the FE analysis for a friction coefficient of 0.2.

**Figure 11:**
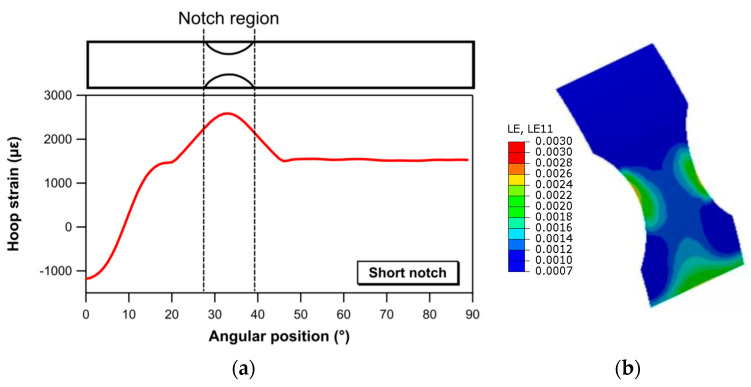
(**a**) Hoop strain as a function of the angular position and (**b**) hoop strain distribution within the notch region for the short notch specimen for an applied load of 5300 N.

**Figure 12:**
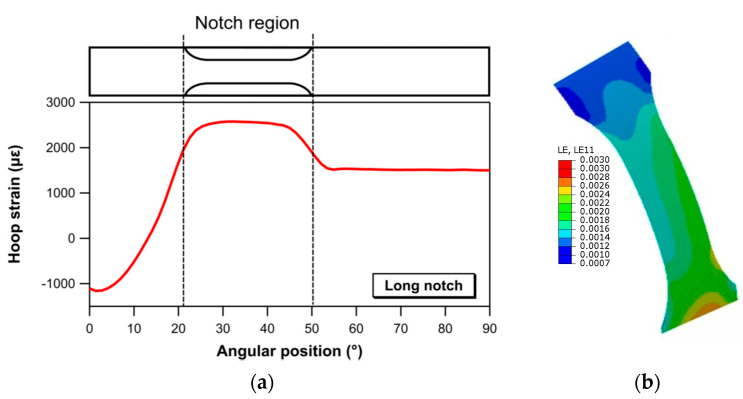
(**a**) Hoop strain as a function of the angular position and (**b**) hoop strain distribution within the notch region for the long notch specimen for an applied load of 5300 N.

Maps and profiles of hoop strain extracted by FE analyses for the two notched geometries are presented in Figure 11 (short notch) and Figure 12 (long notch). The strain values reported in Figure 11 and Figure 12 are calculated for an applied load of 5300 N, chosen as a value below the load corresponding to the initiation of damage in the material. Both the maps and the profiles show that the short notch geometry introduces a strongly uneven strain field, with maximum strain values localized at a specific angular location corresponding to the narrower section. In contrast, a region with a reasonably uniform strain field over a sufficiently extended circumferential length (about 20 mm, as seen in the strain profile of Figure 12) is observed for the long notch configuration. The outcomes of this comparison, which may partly explain the difference in strength observed between the short notch and long notch specimens, emphasize the need for careful consideration of the geometry chosen for area reduction when assessing the strength properties of composite rings.

## 6. Conclusions

The adoption of composite insulators for the construction of high voltage circuit breakers requires reliable and easily applicable procedures for the experimental evaluation of the strength of the component. The split-disk testing method, as described in various international standards, provides a straightforward and cost-effective approach for assessing the hoop strength of composite cylindrical vessels. The study investigated experimentally and numerically some issues related to the application of the testing method that, according to the authors, require some further scrutiny. The main outcomes of the study can be summarized in the following points.

- Significant bending and stress concentration effects occur in the unsupported portion of the specimen at the gap between the semi-disks. The introduction of an area reduction via a notch is necessary to enforce failure in a region of the specimen not affected by these stress disturbances.
- The friction between the specimen and the semi-disks has a noteworthy effect on the hoop load applied to the specimen wall by the split-disk fixture. Large friction forces between the semi-disks and the specimen result in hoop load values greatly dependent on the angular position and significantly lower than those estimated in accordance with the ASTM and EN standards. In contrast, an almost constant distribution of the hoop load, which is thus representative of the real operating conditions of the pressurized insulator, can be achieved by careful lubrication of the disk/specimen contact surface.
- The FE analyses show that the notch geometry specified by the ASTM and EN standards (short notch) for introducing an area reduction is not capable of generating a strain field with a uniform distribution. Conversely, a rather homogeneous strain distribution is achieved on the specimen with the notch geometry proposed in this study (long notch), which is characterized by a 20 mm long gauge length.
- The results of the experimental tests and of the FE analyses show that the hoop strength evaluated on the short notch specimens is higher than that determined on the long notch specimens. These results suggest that special caution must be taken when evaluating the hoop strength of the composite vessel using specimens with notch geometries that induce markedly non-uniform strain distributions.

## Figures and Tables

**Table 1:** Average strength of the three types of specimens: unnotched, short notch, and long notch.

	Unnotched	Short Notch	Long Notch
Hoop strength (MPa)	190	290	238
Standard deviation (MPa)	8.6	4.5	13.6
Number of tested samples	6	10	24

**Table 2:** Elastic properties and Hashin’s damage model parameters employed in the FE model.

Elastic Properties		Hashin Damage Model Parameters	
$\mathrm{Young}’s\;\mathrm{modulus}\;\mathrm{in}\;\mathrm{fiber}\;\mathrm{direction}\;(E_{x}$)	38.6 GPa	$\mathrm{Longitudinal}\;\mathrm{tensile}\;\mathrm{strength}\;(X_{t}$)	950 MPa
$\mathrm{Young}’s\;\mathrm{modulus}\;\mathrm{in}\;\mathrm{transverse}\;\mathrm{direction}\;(E_{y}$)	6.0 GPa	$\mathrm{Longitudinal}\;\mathrm{compressive}\;\mathrm{strength}\;(X_{c}$)	500 MPa
$\mathrm{Young}’s\;\mathrm{modulus}\;\mathrm{in}\;\mathrm{thickness}\;\mathrm{direction}\;(E_{z}$)	6.0 GPa	$\mathrm{Transverse}\;\mathrm{tensile}\;\mathrm{strength}\;(Y_{t}$)	50 MPa
$\mathrm{Shear}\;\mathrm{modulus}\;(G_{xy}=G_{yz}=G_{xz}$)	2.0 GPa	$\mathrm{Transverse}\;\mathrm{compressive}\;\mathrm{strength}\;(Y_{c}$)	320 MPa
Poisson’s coefficient	0.27	$\mathrm{Longitudinal}\;\mathrm{shear}\;\mathrm{strength}\;(S_{l}$)	100 MPa
		$\mathrm{Transverse}\;\mathrm{shear}\;\mathrm{strength}\;(S_{t}$)	100 MPa
		$\mathrm{Longitudinal}\;\mathrm{tensile}\;\mathrm{fracture}\;\mathrm{energy}\;(G_{f,t}$)	20 N/mm
		$\mathrm{Longitudinal}\;\mathrm{compressive}\;\mathrm{fracture}\;\mathrm{energy}\;(G_{f,c}$)	20 N/mm
		$\mathrm{Transverse}\;\mathrm{tensile}\;\mathrm{fracture}\;\mathrm{energy}\;(G_{m,t}$)	1.2 N/mm
		$\mathrm{Transverse}\;\mathrm{compressive}\;\mathrm{fracture}\;\mathrm{energy}\;(G_{m,c}$)	1.2 N/mm

## Data Availability

The data presented in the paper are available upon request.
